# RNA-seq Analysis Reveals Gene Expression Profiling of Female Fertile and Sterile Ovules of *Pinus* *Tabulaeformis* Carr. during Free Nuclear Mitosis of the Female Gametophyte

**DOI:** 10.3390/ijms19082246

**Published:** 2018-08-01

**Authors:** Yang Yao, Rui Han, Zaixin Gong, Caixia Zheng, Yuanyuan Zhao

**Affiliations:** Laboratory of Woody Plants Growth and Development, College of Biological Sciences and Technology, Beijing Forestry University, Beijing 100083, China; yaoyang19880504@126.com (Y.Y.); hanrui@bjfu.edu.cn (R.H.); gongzaixin@bjfu.edu.cn (Z.G.); zhengcx@bjfu.edu.cn (C.Z.)

**Keywords:** conifer, female gametophyte development, megagametogenesis, ovule abortion, transcriptome sequencing

## Abstract

The development of the female gametophyte (FG) is one of the key processes of life cycle alteration between the haploid gametophyte and the diploid sporophytes in plants and it is required for successful seed development after fertilization. It is well demonstrated that free nuclear mitosis (FNM) of FG is crucial for the development of the ovule. However, studies of the molecular mechanism of ovule and FG development focused mainly on angiosperms, such as *Arabidopsis thaliana* and further investigation of gymnosperms remains to be completed. Here, Illumina sequencing of six transcriptomic libraries obtained from developing and abortive ovules at different stages during free nuclear mitosis of magagametophyte (FNMM) was used to acquire transcriptome data and gene expression profiles of *Pinus tabulaeformis*. Six cDNA libraries generated a total of 71.0 million high-quality clean reads that aligned with 63,449 unigenes and the comparison between developing and abortive ovules identified 7174 differentially expressed genes (DEGs). From the functional annotation results, DEGs involved in the cell cycle and phytohormone regulation were highlighted to reveal their biological importance in ovule development. Furthermore, validation of DEGs from the phytohormone signal transduction pathway was performed using quantitative real-time PCR analysis, revealing the dynamics of transcriptional networks and potential key components in the regulation of FG development in *P. tabulaeformis* were identified. These findings provide new insights into the regulatory mechanisms of ovule development in woody gymnosperms.

## 1. Introduction

Seeds are generated from ovules after fertilization. Ovules, which generally include the nucellus, the female gametophyte (FG), the integuments and the funiculus, are female reproductive organs that provide support and protection for the FG. As the core of the ovule, the FG is a structure that gives rise to an egg cell for fertilization. Due to the importance of the FG in ovule development, particularly in seed production, the developmental process has been well studied [1,2].

In angiosperms, the FG arises from the archespore in the nucellus. The archespore then differentiates into the megaspore mother cell (MMC), which undergoes meiosis to generate four megaspores. Only one of them, the functional megaspore, undergoes three rounds of nuclear mitosis to produce a coenocyte with eight nuclei. After nuclear migration, polar nuclei fusion and cellularization, the FG finally becomes a seven-celled structure [3]. It is likely that the development of gymnosperm ovules is similar to that of *Arabidopsis* from the sporogonium to a functional megaspore [3]. Then, multiple free nuclei form through nuclear divisions in the functional megaspore (free nuclear mitosis of the megagametophyte, FNMM) and undergo cellularization to generate the cellular FG in gymnosperms [4]. The process of free nuclear mitosis (FNM) is indispensable for ovule development and the arrest or redundancy of FNM leads to the sterility of the FG. Therefore, it is important to study the FNM process in ovule development.

Over the past decades, our understanding of the regulation of FNMM has expanded rapidly. Accumulating evidence shows that the FNM is ensured by proper mitosis, cellular metabolism and developmental regulation [1]. Genetic studies [5,6] have revealed some functional genes that regulate FNMM, such as genes involved in the cell cycle and phytohormone response. For example, many FG mutations, which were identified through a distorted Mendelian segregation screen, display mitotic arrest of the FNM [7], indicating that the progression of the mitotic cycle is crucial for the formation of a functional gametophyte. Also, identification of these genes shows the importance of cell cycle progression being regulated during female gametogenesis in plants [8,9]. To date, much of our understanding of FG development during FNM is based on studies on *Arabidopsis*; however, gene expression and its possible role remain largely unknown in gymnosperms, particularly, during the FNM.

The *Pinus tabulaeformis* Carr. is an indigenous conifer species that is essential in afforestation in China. The propagation of *P. tabulaeformis* mainly relies on seeds but excessive empty seed formation causes serious yield losses. A previous study reported that the female sterile line (SL) in the *P. tabulaeformis* seed orchard in Xingcheng, Liaoning, China, had normal appearance of the cone but it did not produce seeds because of FNMM termination at an early stage, leading to abortion of the ovule [10]. To understand the FNMM process, we have systematically carried out research on factors underlying the sterility of the mutated line, such as identification of cytological characteristics of SL ovules during FNM process [11], developmental stages [11], metabolism, nutrients [10], phytohormones [12] and proteomes [13]. On the basis of accumulation of our investigations over the years, this study is part of a series examining of the FG development of *P. tabulaeformis*. Here, we profiled global mRNA expression in *P. tabulaeformis* ovules to increase our understanding of the regulatory mechanisms underlying FG development. We also confirmed the gene expression for DEGs from the phytohormone signal transduction pathway and identified potential functional genes in FG development, thereby providing new insights into the molecular mechanisms that regulate FG development in this coniferous species.

## 2. Results

### 2.1. Sequencing, Read Assembly and Global Data Analysis

In the present study, to obtain development-related DEGs of *P. tabulaeformis* ovules, female fertile and sterile ovules at different developmental stages in the FNM process were used in RNA-seq analysis. The developmental stages were selected based on former researches [11], including stage 1 (early February, early period of FNM process), stage 2 (early March, middle period of FNM process) and stage 3 (early April, late period of FNM process). Female fertile line (FL) ovule samples at stages 1, 2 and 3 were marked by FNM1-FL, FNM2-FL and FNM3-FL, respectively, while female sterile line (SL) ovule samples at stages 1, 2 and 3 were marked by FNM1-SL, FNM2-SL and FNM3-SL, respectively. Six RNA-seq libraries were constructed using total RNA from ovule samples.

For an overview of the *P. tabulaeformis* ovule transcriptome, as shown in Table 1, a total of 14.34 G nucleotides was generated with an average Q20 percentage of 97.35% on the Illumina HiSeq™ 2000 platform. The sequencing data were submitted to the NCBI Sequence Read Archive (SRA) database (https://www.ncbi.nlm.nih.gov/sra/) with accession numbers SRR7076840 to SRR7076845. Based on the high-quality reads, a total of 63,449 unigenes were assembled by the Trinity program with a mean length of 741 bp. The summary of unigene assembly is shown in Table 2.

**Figure 1:**
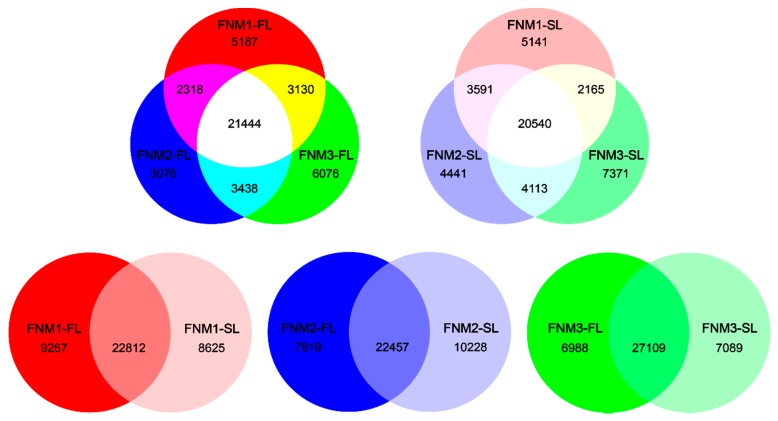
Genes expressed in the female fertile line (FL) and sterile line (SL) ovules at three developmental stages.

Given the assembled sequences, a total of 63,449 genes (32,079 in FNM1-FL, 30,276 in FNM2-FL, 34,088 in FNM3-FL, 31,437 in FNM1-SL, 32,685 in FNM2-SL, 34,189 in FNM3-SL) were generated from six libraries. The summary of gene co-expression was performed as shown in Figure 1. In FL, 21,444 genes were expressed at all three ovule development stages. At the same time, 2318 genes were co-expressed in FNM1-FL and FNM2-FL, 3438 genes were co-expressed in FNM2-FL and FNM3-FL and 3130 genes were co-expressed in FNM1-FL and FNM3-FL. In addition, 5187 (FNM1-FL), 3076 (FNM2-FL) and 6076 (FNM3-FL) genes were specifically expressed in the respective developmental stages. We also found that, in SL, 20,540 genes were expressed in all of the stages. In total, 3591 genes were co-expressed in FNM1-SL and FNM2-SL, 4113 genes were co-expressed in FNM2-SL and FNM3-SL, 2165 genes were co-expressed in FNM1-SL and FNM3-SL. 5141 (FNM1-SL) and 4441 (FNM2-SL) and 7371 (FNM3-SL) genes were specifically expressed in each respective developmental stage. Furthermore, 22,812 genes were co-expressed in FNM1-FL and FNM1-SL, 22,457 genes were co-expressed in FNM2-FL and FNM2-SL and 27,109 genes were co-expressed in FNM3-FL and FNM3-SL. Specifically, FL and SL ovules had some uniquely expressed genes at each developmental stage. At FNM1, the numbers of preferentially expressed genes were 9267 (FNM1-FL) and 8625 (FNM1-SL). At FNM2, the numbers of preferentially expressed genes were 7819 (FNM2-FL) and 10,228 (FNM2-SL). At FNM3, the numbers of preferentially expressed genes were 6988 (FNM3-FL) and 7089 (FNM3-SL).

### 2.2. Gene Annotation and Functional Classification

For annotation of generated unigenes, alignment searches were conducted against public bioinformatics databases. As shown in Table 3, among these unigenes, 49.7% (31,321/63,449) had hits to proteins in public databases, including the NCBI non-redundant (NR) database, Swiss-Prot protein database, Gene Ontology (GO) and Kyoto Encyclopedia of Genes and Genomes (KEGG). The results indicated that of 63,449 unigenes, 30,766 (48.5%) had strong similarity to proteins in the NR database. Similarly, up to 35,782 unigenes (35.4%) had Swiss-Prot annotation.

To further evaluate the function distribution of our transcriptome, we used GO annotation to classify the predicted *P. tabulaeformis* genes. Based on sequence homology, 25,246 (39.8%) unigenes were categorized to GO terms. In each of the main GO classifications (‘cellular component,’ ‘molecular function’ and ‘biological process’), the ‘cell part,’ ‘binding’ and ‘cellular process’ terms, respectively, were dominant. Also, we noticed a high representation of unigenes from the ‘cell,’ ‘catalytic activity’ and ‘metabolic process.’ The GO analysis indicated that the identified genes are associated with various biological processes.

The KEGG database contains the pathway and network of molecular regulation in cells, linking genes and gene products in the pathway. Based on a comparison against the KEGG database using the BLASTX program, 6583 (10.4%) unigenes of *P. tabulaeformis* ovules were mapped into 119 pathways (Table S1). The maps with the highest unigene representation were ‘ribosome pathway’ (ko03010) with 361 counted, followed by ‘oxidative phosphorylation’ (ko00190), ‘protein processing in endoplasmic reticulum’ (ko04141), ‘glycolysis/gluconeogenesis’ (ko00010), ‘starch and sucrose metabolism’ (ko00500), ‘spliceosome’ (ko03040) and ‘RNA transport’ (ko03013). These annotations provide a valuable resource for investigating the biological pathways and gene functions involved in FNM in ovule development.

### 2.3. Differentially Expressed Genes (DEGs) in Comparisons between Fertile and Sterile Ovules during FNMM

To better understand the gene expression differences between fertile and sterile ovules in the FNM process, the DEGs were gathered by using the reads per kb per million reads (RPKM) method to calculate and compare the expression level. The DEGs with a false discovery rate (FDR) <0.01 and a fold change of differential expression >2 were confirmed as significant differences. A total of 7174 genes were found differentially expressed at the three developmental stages (shown in Table 4). Although a high number of DEGs were found at all stages, the highest number was found at FNM2, suggesting the evident difference in the transcriptional level at FNM2. The DEGs at FNM1 showed the beginning of the cell fate divergence, while those at FNM3 corresponded to the transcriptomic difference between the maturation and degradation of FG. In addition, the complete list of DEGs between FL and SL ovules is shown in Table S2, including overlapping DEGs in all stages or any two stages.

**Figure 2:**
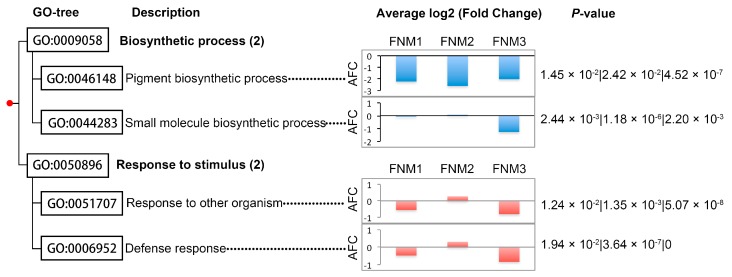
Significantly enriched GO terms in ‘Biological process’ from DEGs in comparison between fertile and sterile ovules in *Pinus tabulaeformis* during free nuclear mitosis. The significance of effects was determined by examining enrichment of gene ontology (GO) terms associated with differentially expressed genes (DEGs) versus the respective GO terms in the whole genome distribution using a hypogeometric test and a threshold of the Benjamini and Hochberg false discovery rate (FDR) corrected to *p* < 0.05. The *y*-axes indicate the value of average log2 (fold change) (AFC), which suggest the fold change in expression level between the FL and SL for all of the genes that belong to the respective GO term. Bars in a specific GO term represent AFC at different developmental stages. Other ontologies are shown in supporting information Table S3.

To gain further insights into the biological processes associated with the observed temporal changes, we also performed GO enrichment analysis for DEGs in comparison between FL and SL ovules at each developmental stage (Table 5). We then identified a core set of biological processes which were significantly enriched during the three studied developmental stages, including ‘defense response’ (GO:0006952), ‘small molecule synthetic process’ (GO:0044283), ‘response to other organism’ (GO:0051707) and ‘pigment biosynthetic process’ (GO:0046148) (Figure 2). The ‘defense response’ (GO:0006952) category includes genes associated with restriction or prevention with the respect to injury or infection, suggesting the communication between stress signals and perception in the ovule developmental procedure. The majority of genes in the ‘pigment biosynthetic process’ (GO:0046148) category showed down-regulated trends throughout the studied developmental stages. Genes in other processes represented initial and final upregulation and were downregulated in the FNM2 stage. The ‘biosynthetic process,’ containing the ‘small molecule synthetic process’ (GO:0044283) and ‘pigment biosynthetic process’ (GO:0046148), suggests irregular gene expression in synthetic reactions in the FNM process. Then, the GO terms enriched specifically in one or two developmental stages were studied (Table S3). Notably, twelve enriched categories associated with stimulus were found throughout the three studied stages, including both stress and phytohormone. Six stage-specific ontologies representing morphogenesis and development were enriched. The last developmental stage was specifically enriched in ontologies associated with secondary metabolism and stimulus and most of the ontologies specifically enriched in this stage are upregulated in SL. In addition, many DEGs from comparison between FL and SL ovules at FNM2 stage were annotated to GO term ‘regulation of cell death,’ which was specially enriched at the FNM2 stage but not enriched at other two developmental stages.

**Figure 3:**
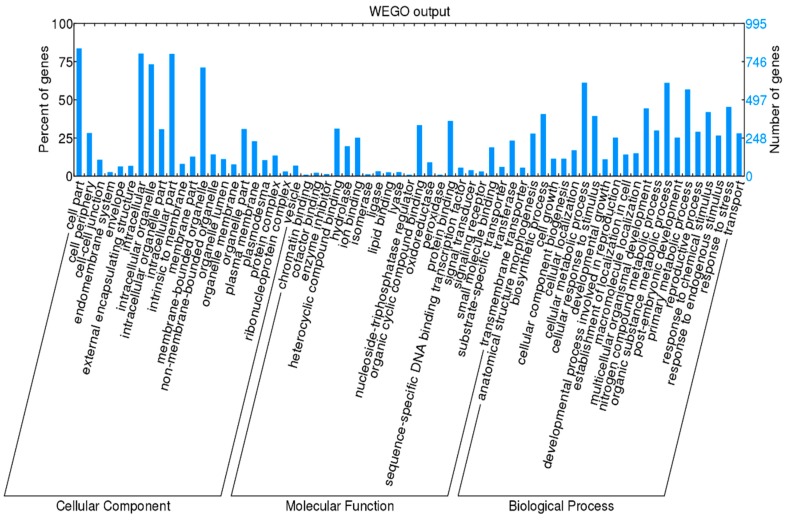
GO analysis for DEGs preferentially expressed in FL ovules. DEGs were grouped to the secondary classification of hierarchical GO terms.

In our study, abnormal FG development was the main factor responsible for the ovule abortion. To acquire the preliminary insight into gene regulation of ovule development, the scope of putative FG or ovule specific genes among all DEGs were narrowed down based on former research [14]. The selected genes were preferentially expressed in FL ovules (listed in Table S4). GO annotation was carried out to further evaluate the gene function category (Figure 3). In the biological process category, many genes were involved into processes of development, growth and reproduction and these genes might be candidate of the FG or ovule specific genes.

**Figure 4:**
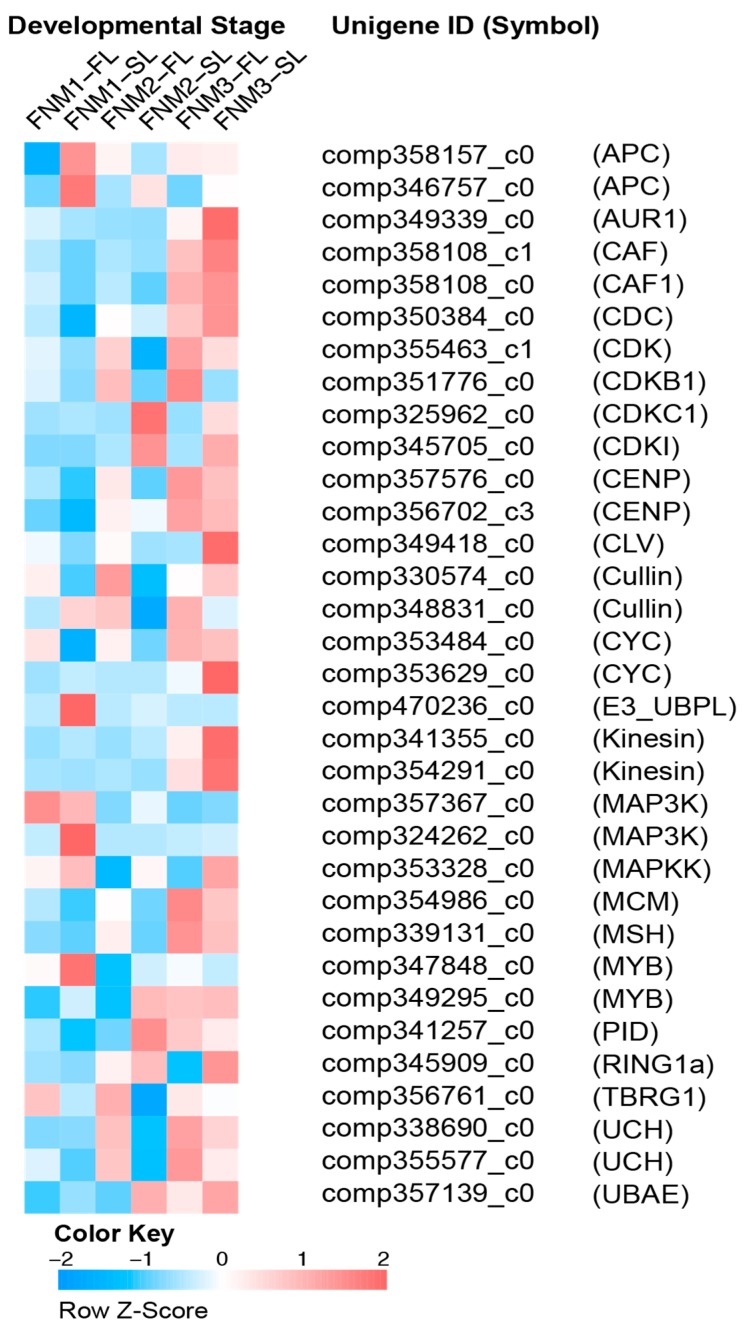
Specific temporal changes of cell cycle-associated genes during FNM. For the full names and expression and annotation data of the genes, see Tables S2 and S5. Data for the gene expression level were normalized to *Z*-score.

The clear indication that the whole FNM process corresponds to the continuous caryokinesis prompted us to more closely inspect genes of known function in the regulation of the cell cycle. Based on functional annotation from Swiss-Prot database, NR database and Gene Ontology, 33 genes encoding key regulators of the cell cycle and mitosis were found. As shown in Figure 4, more genes were up-regulated in FNM1 and FNM2, while most genes in FNM3 were downregulated. Furthermore, more than half of these key regulators were differentially expressed in FNM2.

### 2.4. Expression Patterns of DEGs in FL between Different Developmental Stages during the FNM Process

To acquire the temporal changes of gene expression in FL and SL ovules, genes that were differentially expressed between two developmental stages were analyzed and are listed in Table S5. Between stages FNM1 and FNM2, 2313 (1460 upregulated genes, 853 downregulated genes) DEGs were found in FL and 2229 (1219 upregulated genes, 1010 downregulated genes) DEGs were found in SL. The differences between upregulated and downregulated genes were more significant in FL than in SL. Between stages FNM2 and FNM3, 1678 (740 upregulated genes, 938 downregulated genes) DEGs were found in FL and 2062 (1133 upregulated genes, 929 down-regulated genes) DEGs were found in SL. To explore the transcriptome profile of developing ovules, we focused on the expression pattern of unigenes in FL ovules.

**Figure 5:**
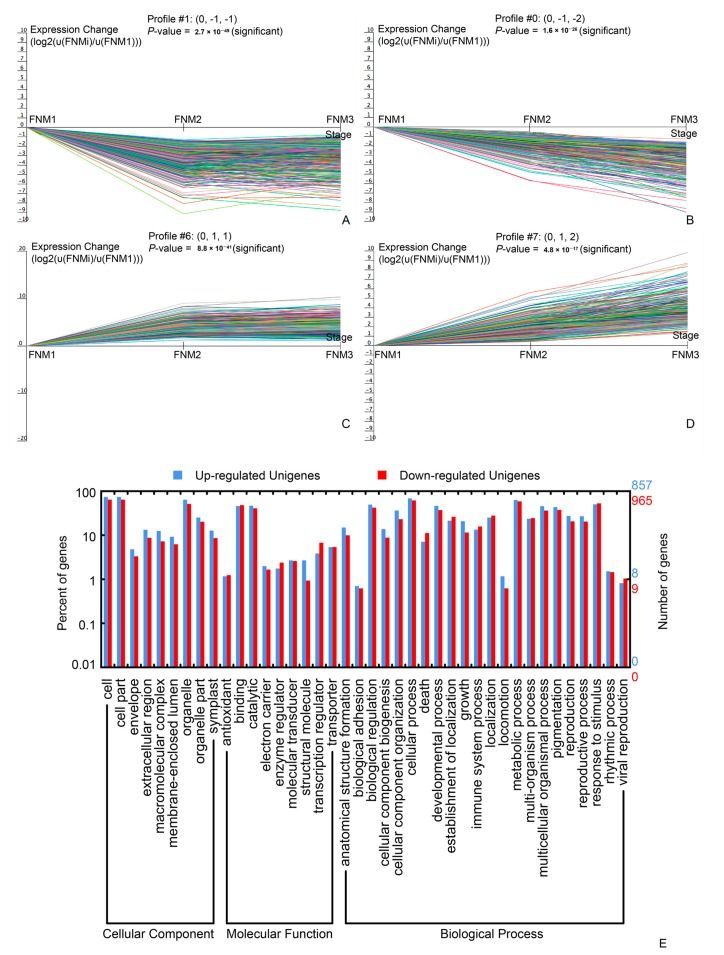
Significant DEG expression profiles (**A**–**D**) and their GO classification (**E**) in female fertile ovules. Profile 1 (**A**) and profile 0 (**B**) indicates a down-regulated trend and profile 6 (**C**) and profile 7 (**D**) indicates an up-regulated trend from FNM1 to FNM3 stage. GO analysis for these unigenes was shown (**E**). The down-regulated unigenes are union of profile 0 and profile 1. The up-regulated unigenes are union of profile 6 and profile 7.

To examine the expression profile of DEGs in ovule development, the expression data υ of FL (in FNM1, FNM2 and FNM3) were normalized to 0, log_2_ (υ_FNM2_/υ_FNM1_), log_2_ (υ_FNM3_/υ_FNM1_). DEGs could be clustered into 8 profiles by Short Time-series Expression Miner (STEM) software in which 1822 were clustered into 4 profiles (*p* < 0.05), containing two downregulated patterns (profile 1 and 0) and two upregulated patterns (profile 6 and 7) (Figure S1). Profile 1 and 0 included 635 and 330 DEGs, respectively and profile 6 and 7 contained 562 and 295 DEGs, respectively. Next, the DEGs within the up- and down-regulated cluster groups were subjected to GO-term analysis. They were classified into 3 main categories including ‘cellular component’, ‘molecular function’ and ‘biological process’. In the ‘cellular component’ category, most of the up- and down-regulated DEGs were classified into ‘cell’ (GO:0005623), ‘cell part’ (GO:0044464) and ‘organelle’ (GO:0043226). In the ‘molecular function’ category, a high number of upregulated as well as downregulated DEGs was categorized as ‘binding’ (GO:0005488) and ‘catalytic activity’ (GO:003824). In the ‘biological process’ category, ‘cellular process’ (GO:0009987) and ‘metabolic process’ (GO:0008152) were the top abundant subcategories (Figure 5).

Then, DEGs were subjected to KEGG pathway enrichment analysis: 19.0% (482/2555) of the DEGs could be annotated. The 10 top KEGG pathways with the highest representation of the DEGs are shown in Table 6. The metabolic pathways (ko01100), biosynthesis of secondary metabolites (ko01110), biosynthesis of antibiotics (ko01130), microbial metabolism in diverse environments (ko01120), phenylpropanoid biosynthesis (ko00940), plant hormone signal transduction (ko04075), carbon metabolism (ko01200), DNA replication (ko03030), cell cycle (ko04111) and biosynthesis of amino acid (ko01230) pathways were enriched. In the plant hormone signal transduction pathway, the 7 unigenes among 113 DEGs (7.19%) in profile 1 and 3 unigenes accounting for 4.76% of 63 in profile 0 were annotated, whereas in profile 6 and 7, only 4 unigenes accounting for 4.08% of 98 DEGs and 0 of 64 DEGs were annotated to this pathway.

**Figure 6:**
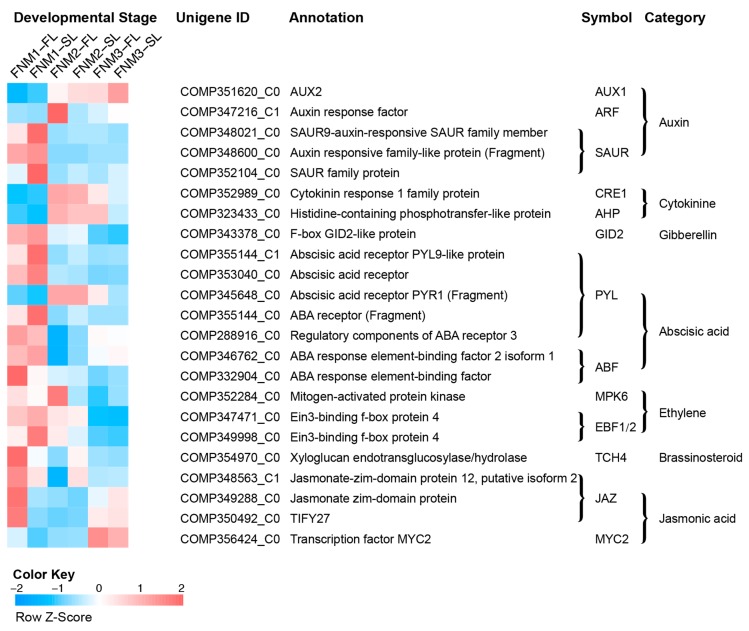
Heat map diagram of expression levels of DEGs annotated in the plant hormone signal transduction pathways analyzed by KEGG. Data for the gene expression level were normalized to *Z*-score.

The number of DEGs involved in the plant hormone signal transduction pathway during the FNM process was listed in Table S6. A total of 23 DEGs were annotated in the plant hormone signal transduction pathways, including auxin, cytokinin, gibberellin, abscisic acid (ABA), ethylene, brassinosteroid and jasmonic acid. As shown in Figure 6, we identified 5 differentially expressed genes encoding auxin signaling components and 3 of them encoding Small auxin-up RNA (SAUR) family proteins were downregulated (profile 1). In the cytokinin signaling pathway, 1 unigene encoding cytokinin response 1 (CRE1) protein showed upregulated trends. In the gibberellin signal transduction pathway, 1 unigene encoding glucose-induced degradation protein (GID2) was identified as a downregulated profile. In the abscisic acid signal transduction pathway, 4 out of the 7 DEGs were clustered into profile 0 or 1, showing downregulated trends. They encode abscisic acid receptors PYR/PYL family protein and ABA responsive element binding factor (ABF). Unigene COMP345648_C0 encoding abscisic acid receptor showed an upregulated trend. In the ethylene signal transduction pathway, 3 unigenes were also identified as DEGs, including 1 unigene encoding mitogen-activated protein kinase 6 (MPK6) and 2 encoding EIN3-binding F-box protein (EBF1/2). In the brassinosteroid signal transduction pathway, 1 unigene encoding xyloglucosyl transferase TCH4 was annotated to profile 1, showing downregulated trends. In the jasmonic acid signal transduction pathway, 3 unigenes encoding the jasmonate ZIM domain-containing protein (JAZ) and 1 encoding transcription factor MYC2 were found to be differentially expressed and 1 unigene was identified as having a downregulated profile. Therefore, the changes in expression patterns of specific transcripts suggest the necessity of phytohormone regulation in ovule development. These results documented changes in the expression of genes regulating the level of phytohormone that may be involved in free nuclear mitosis.

Interestingly, shown in Table 6, most DEGs involved in the energy metabolism, including ‘carbon metabolism’ and ‘biosynthesis of amino acid’ indicated upregulated trends in FNM process in FL ovules. A total of 40 DEGs were selected by KEGG pathways and listed in Table S7. Genes encoding NAD-dependent malic enzyme, serine hydroxymethyltransferase, 6-phosphofructokinase, ribulose bisphosphate carboxylase, phosphoglycerate kinase, formate dehydrogenase, glyceraldehyde-3-phosphate dehydrogenase, transketolase, fructose-bisphosphate aldolase, malate synthase, S-adenosylmethionine synthase, glutamine synthetase, phosphoglycerate kinase, aminotransferase were downregulated from FNM1 to FNM2, FNM2 to FNM3, or FNM1 to FNM3, respectively, while genes encoding fructose-1,6-bisphosphatase, alcohol dehydrogenase, shikimate kinase and argininosuccinate synthase showed upregulated trends in the FNM process in temporal order.

### 2.5. Verification of the Gene Expression Profile by qRT-PCR

**Figure 7:**
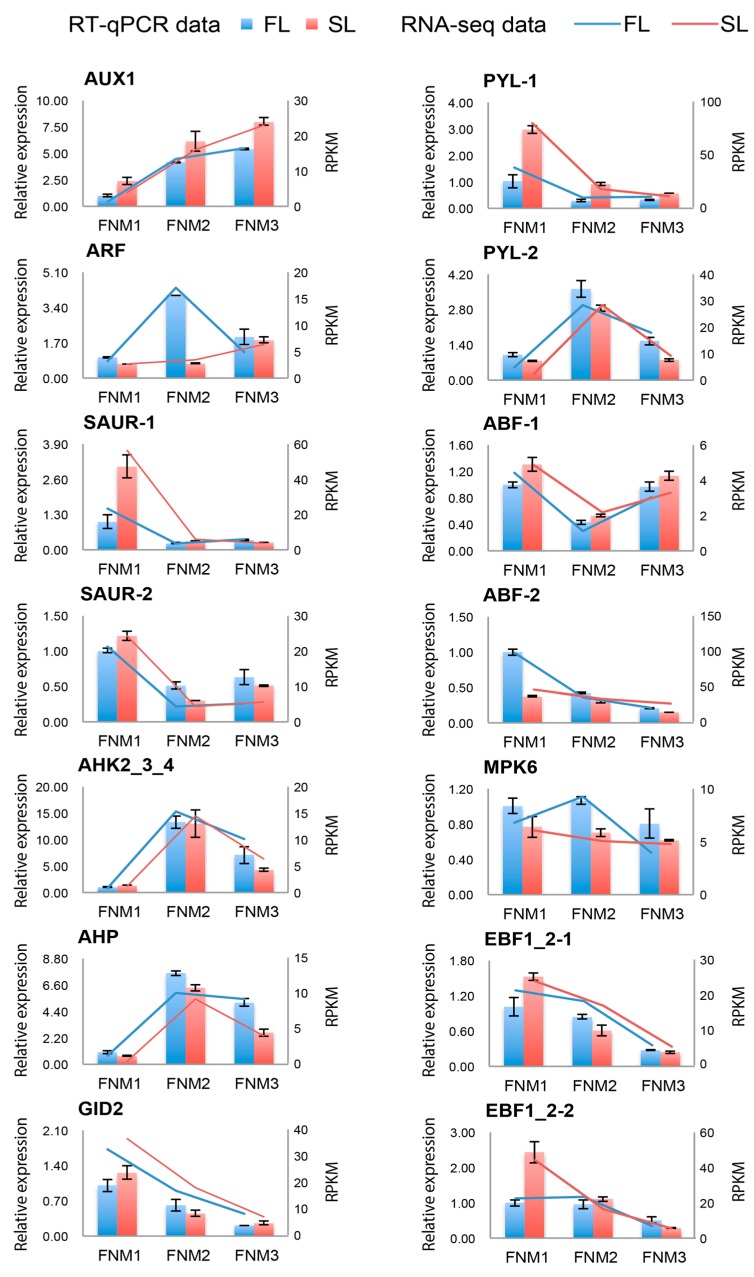
Candidate unigenes expression levels revealed by qRT-PCR and RNA-seq. Data from qRT-PCR are the means of three technical replicates and bars represent SE. RPKM values from RNA-seq were generated from pooled ovule samples.

**Figure 8:**
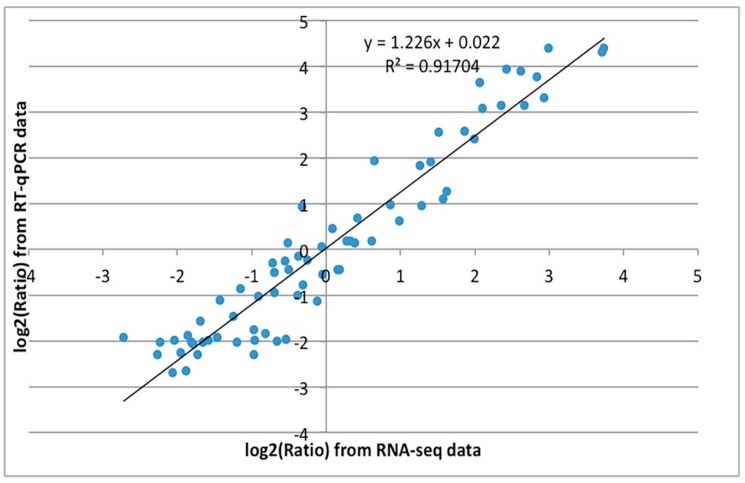
Coefficient analysis of fold change data between qRT-PCR and RNA-seq. 14 unigenes were selected for qRT-PCR. Data indicating the relative transcript level from qRT-PCR are the means of three replicates. Scatterplots were generated by the log2 expression ratios from RNA-seq (*x*-axis) and RT-qPCR (*y*-axis).

To confirm the transcriptomic analysis results, DEGs were selected for quantitative real-time PCR (qRT-PCR) validation using the same type of samples compared with formerly used samples in RNA-seq analysis, including genes encoding auxin influx carriers AUX1/LIKE-AUX1 (AUX/LAX) protein, auxin response factor (ARF), small auxin-up RNA (SAUR), Arabidopsis histidine kinases (AHK), histidine phosphotransfer protein (AHP), glucose-induced degradation protein (GID), pyrabactin resistance 1 protein (PYR), ABA responsive element binding factor (ARF), mitogen-activated protein kinase 6 (MPK6) and EIN3 binding F-box protein (EBF). These genes were selected for their key roles in phytohormone signal transduction. The primers of unigenes are shown in Table S8. The expression profiles of the candidate unigenes revealed by qRT-PCR data were consistent with those derived from sequencing (Figure 7). Linear regression analysis of the fold change of the gene expression ratios between RNA-seq and qRT-PCR showed a significantly positive correlation (Figure 8), confirming our transcriptome analysis.

## 3. Discussion

### 3.1. Illumina Paired-End Sequencing, Assembly and Annotation

The transcriptome is used to refer to all RNAs or just mRNA in one cell or a population of cells with temporal and spatial specificity. Therefore, transcriptome analysis is pivotal in revealing the molecular mechanism of biological processes. In the present study, we sampled the transcriptome of *P. tabulaeformis* ovules using Illumina paired-end sequencing technology to yield a large number of transcripts. A total of 14.34 G nucleotides was generated and assembled into 63,449 unigenes. The reads from transcriptome sequencing produced much longer unigenes (mean, 741 bp) than those in previous research [15,16]. This increased the sequencing accuracy and depth of the transcriptome from de novo assembly.

Of the *P. tabulaeformis* unigenes, 30,776/63,449 had homologs in the NR databases. Importantly, we assigned a number of these unigenes to a wide range of GO categories, indicating that a wide range of transcripts involved in FNM are represented in the sequence data of this species, reflecting the complexity of the FG development process in woody gymnosperms. Furthermore, most representative unigenes were annotated to specific pathways, such as ‘ribosome’, ‘oxidative phosphorylation’, ‘protein processing in endoplasmic reticulum’ and ‘glycolysis/gluconeogenesis’ using the KEGG database, leading us to conclude that most of the genes we identified are involved in FNM and FG development.

### 3.2. Global Changes of Gene Expression during the FNM Process in Ovule Development

Ovules are female reproductive organs that play a major role in sexual reproduction. Recently, Yang et al. revealed extensive changes in the transcriptome of *Arabidopsis* ovules during the course of ovule development [5] but little attention has been paid to gene expression of FG development in the woody gymnosperm *P. tabulaeformis* during the FNM process. In the present study, using RNA-Seq to examine global gene expression profiles over a time course of FNM in *P. tabulaeformis* ovules, we found the preferential expression of DEGs (|log_2_ (ratio)| > 2) between the FL and SL ovules at three developmental stages, indicating considerable difference in the gene expression of ovules. Since we analyzed the transcriptional profiles following the dynamic process of developing ovules from the FNM1 to FNM3 stage, we obtained more genes with dynamic changes in transcript abundance or those that were only strongly transcribed during the transitions.

### 3.3. Genes Related to Cell Cycle and Mitosis

Progression of the mitotic cycle is important for free nuclear mitosis in megagametogenesis. Many genes encoding components regulating the cell cycle showed different expression patterns during ovule development in FL compared with SL.

Cyclin-dependent kinases (CDKs) make a large contribution to the regulation of cell cycle progression and cell division. Among these proteins, CDKB members are plant-specific kinases and include the CDKB1 superfamily whose kinase activity reaches peak levels during mitosis [17]. Deficiency of CDKB1 and CDKA1 kinases leads to the absence of the egg apparatus within the embryo sac [18]. In the present study, one gene encoding CDKB was upregulated in FNM2-FL and FNM3-FL compared to FNM2-SL and FNM3-SL, respectively, suggesting the difference in gene expression is related to CDK between FL and SL. The CDK inhibitory protein (ICK) negatively regulates CDKs, and, therefore, degradation of ICK is also an essential component of cell cycle regulation [19]. Accordingly, during megagametogenesis in *Arabidopsis thaliana*, precise expression of the gene encoding ICK ensures mitotic cell cycle procession [20]. The present study showed that one gene encoding ICK was downregulated in FNM2-FL and FNM3-FL compared to FNM2-SL and FNM3-SL, respectively. The downregulation of gene expression may lead to the reduction of inhibitors and upregulate the associated CDK protein in FL at the FNM2 and FNM3 stages, contributing to FNM during the developmental stages. It is suggested that the regulator related to CDK may responsible for the process of FNM in FG development. MYB proteins are important transcriptional factors in plants that are involved in various biological processes, including cell cycle, growth and developmental processes. Several members of the MYB family have been proven to be responsible for development of the female gametophyte in *Arabidopsis. FLP* and *MYB88* regulate the FG development and their loss significantly increases the number of ovules [21]. Attenuation of *MYB64* and *MYB119* expression, during transition to the FG5 phase, leads to failure of cellularization of the female gametophyte, resulting in supernumerary nuclei [9], similar to the female gametophyte in gymnosperms. Similarly, in the present study, we found that the free nuclei were unable to conduct mitosis in SL ovules and the expression of 10 *MYB*-like sequences was downregulated in FL ovules compared with SL ovules, suggesting that the MYB family proteins play essential roles in the FNM process and putatively regulate cell cycle gene expression. The minichrome maintenance (MCM) protein family is indispensable in DNA replication and synthesis. The *PRL* gene in *Arabidopsis* encodes a homologue of MCM7, the subunit of a DNA replication licensing factor, which is required in all dividing cells, including FG [22]. In our study, two genes encoding MCM10 and MCM3 proteins, were upregulated in comparison in FNM1-FL versus FNM1-SL and FNM2-FL versus FNM2-SL and the level of gene expression in FL ovules increased from the FNM1 to FNM2 stage, suggesting that MCM proteins function in developing ovules during the FNM process. The anaphase-promoting complex/cyclosome (APC/C) is an essential E3 ubiquitin protein ligase that regulates transition during the mitotic process and is responsible for the degradation of mitotic cyclins [23]. Mutation in the *APC2* gene, which encodes one of APC/C’s subunits, impairs female megagametogenesis [24]. Similarly, the *nomega* mutant embryo sacs, which result in the arrest of mitosis at the two-nucleate stage, are unable to degrade Cyclin B and the gene product of *NOMEGA* shows high homology to the APC6/cell division cycle (CDC) 16 subunit of APC/C [25]. In the present research, two APC-like genes were downregulated in FL ovules compared with SL ovules at the FNM1 stage, implying that the APC/C ubiquitin-mediated proteolysis pathway may play an important role in the beginning of FNM.

### 3.4. Genes Related to Phytohormones

Plant hormones are generated within the plant and serve as signal functions, regulating a wide range of processes. The plant hormone signals are detected and transported to the nucleus by signal transduction to activate gene expression, leading to physiological processes. The DEGs were involved in all of the phytohormone signal transduction pathways. We mainly focused on the expression patterns of auxin- and cytokinin-related genes, which may be responsible for FNM in FG development.

Auxin, a mobile signaling molecule, functions as one of the most critical phytohormones, regulating various processes in plant development. Auxin has been shown as a key regulator in megagametogenesis. Genes related to auxin biosynthesis and auxin influx carriers are revealed, such as *YUCCA1* (*YUC1*) and *AUX1* gene, implying a complicated mechanism of auxin involved in mitotic divisions, cell expansion and patterning during embryo sac development [26].

Auxin biosynthesis plays an important role in ovule development. Data have been confirmed by Note-Wilson [27], who associated ovule loss with a severe reduction in local auxin biosynthesis. Also, the disruption of auxin synthesis by the ectopic expression of the *YUC1* gene impairs cell specificity in the FG [28]. Anthranilate synthase (ASA) is involved in the l-tryptohphan synthesis pathway and is a precursor of IAA. The overexpression of the *α-SUBUNIT OF ANTHRANILATE SYNTHASE* (*OASA1D*) gene in a transgenic rice line increased the level of tryptophan and free IAA [29]. In the present study, one gene encoding ASA was downregulated in FL compared with SL at the FNM3 stage and two *YUCCA* genes were differentially expressed in FNM3-FL compared with FNM3-SL, implying a disorder of auxin biosynthesis in sterile FG.

Increasing evidence indicates that polar auxin transport (PAT) regulates significant processes of growth and development. An auxin gradient along the micropylar-chalazal axis can regulate the specification of cell fate in female gametophyte genesis [28]. A transient application of N-1-naphthylphthalamic acid (an auxin efflux inhibitor) causes a significant loss of ovules [30]. In *Arabidopsis*, the AUX1/LAX auxin transporter family is an influx carrier, while the PIN-FORMED (PIN) auxin protein family is the main efflux carrier [31]. In FG, downregulation of the *PIN1* gene induces mitosis arrest and subsequently, FG sterility [32]. In our study, one gene encoding LAX4 was downregulated in FNM1-FL compared with FNM1-SL and two genes encoding PIN1 were differentially expressed in the FNM2 stage. It is indicated that PAT may be strengthened in SL FG.

Auxin primary response genes include *GH3, SAUR* (*small auxin up RNA*) and *AUX/IAA*. During the process of FNM, genes related to the auxin response were preferentially expressed in different developmental stages. It is suggested that the correct expression of auxin response genes is necessary for the FG development. In our transcriptomic data, one *SAUR* gene was downregulated in FL ovules at all three stages but was upregulated in SL ovules. One *IAA* gene was downregulated in FL at the FNM1 stage. Auxin responsive factors (ARF) bind to the promoter region of the auxin-responsive element to stimulate or repress transcription. Overexpression of microRNA *miR167* leads to the downregulation of its target *ARF6* and *ARF8* genes, inducing the ensuing arrest of FG development [33]. Several *ARF* genes were preferentially expressed in FL and SL. It is hypothesized that the expression variations of auxin-responsive genes between FL and SL may affect the production and distribution of auxin during ovule development, resulting in FG sterility in *P. tabulaeformis*.

However, evidence shows that the gradients in the FG cannot provide sufficient positional instruction for cell specification [34]. This increases the complexity of auxin function in FG development. Although the function of auxin in female gametophyte development is not entirely clear, our study indicates the indispensable role of auxin in the FG formation.

Cytokinin is an essential hormone for cell proliferation and differentiation. Many studies have proven that cytokinin plays an important role during ovule development. Manipulation of cytokinin metabolism affects ovule fertility.

A reduction in the cytokinin gradient in plants induces a decrease in ovule number or female fertility [35]. Cytokinin oxidases/dehydrogenases (CKX) are responsible for the irreversible degradation of cytokinin and the inhibition of hormone concentrations in tissues [36]. Overexpression of cytokinin oxidase reduced the fertility rate [37]. Also, alteration of *CKX* gene expression influences the regulation of cytokinin levels and controls the developmental process of the ovule. The dual mutation of *CKX3* and *CKX5* genes in *Arabidopsis* established supernumerary ovules, further confirming the relationship between cytokinin and ovule development [38]. In the present study, two genes encoding CKX were downregulated in comparison between FL and SL at the FNM2 and FNM3 stages, resulting in less suppression of the cytokinin level and ensuring the FG development. Although the function of cytokinin in ovule development is still not clear, these findings confirm the necessity of cytokinin in ovule development.

In cytokinin signal transduction, the interference of three genes, encoding Arabidopsis histidine kinases (AHK2, AHK3 and AHK4/CRE1), induced a reduction in fertility, phenocopying of the quintuple mutant *ahp1*, *2*, *3*, *4*, *5* and impairment of the cytokinin signaling pathway [39]. On the basis of these studies, we focused on gene expression in cytokinin signal transduction in our research, especially at the FNM1 and FNM2 stages. In the present study, no DEG in a comparison between FL and SL ovules was found in the cytokinin signal transduction pathway but some genes were specifically expressed at different developmental stages. The expression level of genes encoding AHK2/3/4 and AHP was upregulated from the FNM1 to FNM2 stage in FL ovules, suggesting the cytokinin response is enhanced at FNM2 stage. During the period of functional megaspore mitosis, genes related to the cytokinin signaling pathway (*CRE1* and *AHP*) and degradation (*CKX*) were significantly differentially expressed between FNM1 and FNM2 in different lines.

ABA-mediated signaling is believed to be involved in stress response, seed germination and the developmental process of plants. In the ABA biosynthesis pathway, the rate-limiting step is the decomposition of 9-*cis*-epoxycarotenoids and the generation of the ABA precursor xanthoxin and this key process is catalyzed by 9-*cis*-epoxycarotenoid dioxygenase (NCED). In our study, two genes encoding NCED were upregulated in SL ovules compared with FL ovules at the FNM2 and FNM3 stages, suggesting the acceleration of ABA biosynthesis in SL ovules. In the FNM process, the ABA content is higher in SL than in FL at all of the developmental stages [12], which is in agreement with gene expression result in the present study. In the ABA signal transduction pathway, PYL, the receptor for ABA, suppresses protein phosphatase (PP2C) and is the origin of ABA negative regulation [40]. In our study, two genes encoding PYL/PYR were upregulated in FNM1-SL compared with FNM1-FL. Taken together, these data indicate that ABA biosynthesis and signal transduction might be more active in SL ovules.

In addition, an alteration of the phytohormone level not only regulates FG development directly but also controls the cell cycle process and affects FG development indirectly. Auxin induces mitosis cyclin (CYCA, CYCB) in *Arabidopsis* roots and mRNA accumulation of CYCD in the G1 phase. As for the G2/M phase transition, auxin is necessary and regulates the cell cycle process through cyclin degradation by ubiquitin-related protein [41]. Cytokinin induces the synthesis of CYCD3, promoting G1/S phase transition. From the G0 to S phase, exogenous cytokinin activates potential DNA replication and reduces the time span of DNA replication in the S phase. In tobacco, cytokinin activates CDKA kinase in G2/M phase transition [42]. ABA induces ICK and regulates the activity of CDKA in the G1 and G2 phase. It is implied that genes involved in the cell cycle process and phytohormone biosynthesis and signal transduction pathway constitute a complex regulation network to guarantee the FG development; these genes require further exploration.

**Figure 9:**
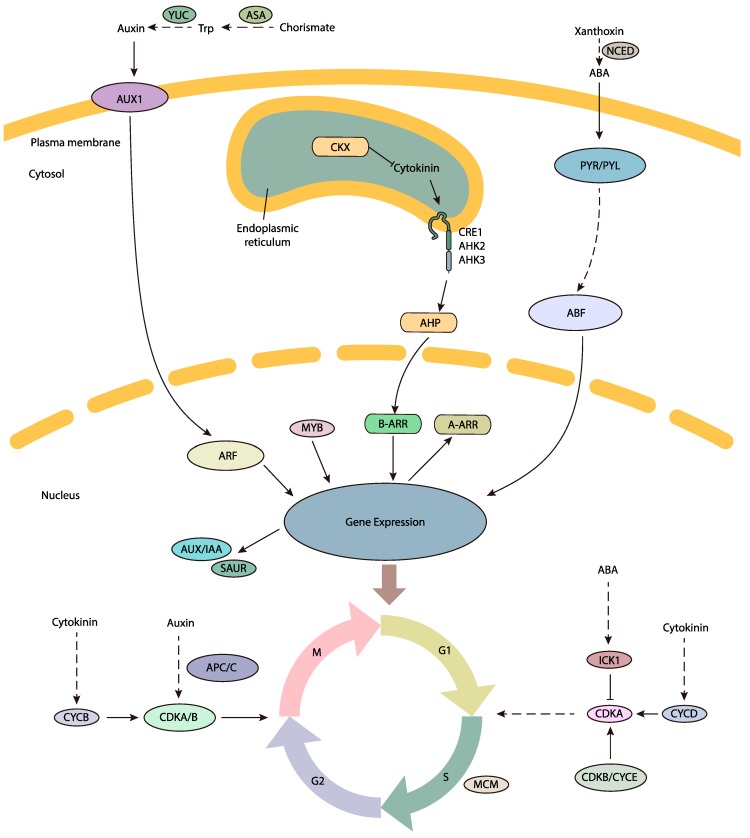
Diagram of the putative regulatory mechanisms of the FNM process. External signals are sensed by FG and may trigger the FNM process. The DEGs, which are found in comparison between developing and abortive ovules, are involved in phytohormone pathways and the cell cycle process. The phytohormone pathway contains the following genes involved in auxin biosynthesis (*YUC* and *ASA*), auxin signal transduction (*AUX1*, *ARF*, *AUX/IAA* and *SAUR*), cytokinin degradation (*CKX*) and signal transduction (*CER1/AHK*, *AHP* and *A-ARR*), ABA biosynthesis (*NCED*) and signal transduction (*PYR/PYL* and *ABF*). The gene expression may also regulate the cell cycle process by up- or down-regulated key genes, such as genes encoding CDKB, ICK1, APC/C and MCM proteins. The line arrow indicates direct interaction between subjects. The dot line arrow suggests the indirect influence or abbreviated multi-step between two subjects. ABF, ABA responsive element binding factor; AHK, Arabidopsis histidine kinase; AHP, Arabidopsis histidine phosphotransfer protein; ARF, auxin response factor; AUX1, AUXIN1; APC/C, anaphase-promoting complex/cyclosome; ASA, anthranilate synthase; CDK, cyclin-dependent kinase; CKX, cytokinin oxidases/dehydrogenases; CRE1, cytokinin response 1; CYC, cyclin; ICK, CDK inhibitory protein; MCM, minichrome maintenance; NCED, 9-*cis*-Epoxycarotenoid dioxygenase; PYL, PYR1-Like protein; PYR, pyrabactin resistance 1 protein; SAUR, small auxin-up RNA; YUC, YUCCA.

In summary, we reported a comprehensive *P. tabulaeformis* ovule transcriptome dataset generated by de novo assembly of next-generation sequencing data, allowing gene expression changes between developing and abortive ovules to be compared. The DEG analysis revealed that two groups of genes were putatively involved in the FNM process, including cell cycle regulation and phytohormone pathways (shown in Figure 9). DEGs encoding CDKB, ICK, MCM, MYB and the APC/C protein were preferentially expressed in developing ovules, securing the cell cycle process. DEGs encoding ASA, YUC, AUX1, ARF, AUX/IAA and SAUR in the auxin pathway, CKX, CRE/AHK and AHP in the cytokinin pathway and NCED and PYR/PYL in the ABA pathway may potentially induce the phytohormone regulation and trigger the FNM process. In addition, phytohormones might regulate cell cycle process and indirectly regulate the FNM, constructing a comprehensive regulation network for the FNM process. The *P. tabulaeformis* transcriptome data is a valuable resource for future genomic studies and will be helpful for researchers working on other closely related species. The differentially expressed genes dataset will also provide useful candidate genes for functional analysis of ovule development in woody gymnosperms.

## 4. Materials and Methods

### 4.1. Sample Collection

Our research group previously found that the FL ovule and SL ovule were genetically closely related through a DNA marker technique [43]. Thus, the FL and SL ovules can be used to explore the mechanism of ovule development in *P. tabulaeformis.* Ovules from a female sterile line (SL) and a fertile line (FL) were used in this study. Then, three SL trees and FL trees were selected in a *Pinus tabulaeformis* seed orchard in Xingcheng, Liaoning Provence, China (40°44′ N, 120°34′ E, 100 m above sea level). The selected trees were growing in identical habitats. Biennial cones of similar size, whose ovules were in stage 1 (early February), stage 2 (early March) and stage 3 (early April) of FNMM, were gathered from the middle of the crown of every tree. The collection was permitted by the Forestry Administration of Xingcheng. Scales of the same size in the middle of the cones were stripped. Then, same-sized ovules were isolated from the placenta of scales by a microdissection method under a dissection microscope and their bracts and scales were carefully removed. Each sample included ovules from five cones. These samples were immediately transferred into liquid nitrogen overnight and were then stored at −80 °C.

Based on the correspondence between the morphological features and developmental stages of the FG, the ovules were collected at three stages: ovules containing a functional megaspore with dozens of free nuclei (stage 1, FNM1), hundreds of free nuclei (stage 2, FNM2) and approximately 1000 free nuclei (stage 3, FNM3).

### 4.2. RNA Extraction, Library Construction and RNA-seq

Total RNA of the ovules was extracted using Plant RNA Assistant Kit (Kebaiao, Beijing, China) according to the manufacturer’s instruction. The quantity and quality of RNA samples were determined by Nanodrop 2000 spectrophotometer (Thermo Scientific, MA, USA) and gel electrophoresis. For each sample, identical quantity of total RNA from three biological replicates was pooled for library construction before high-throughput sequencing. Briefly, mRNA was isolated with Oligo (dT) cellulose, fragmented by using fragmentation buffer and reverse transcripted with random hexamer primers. Second-strand cDNA were synthsized by DNA polymerase I and Rnase H. Then the cDNA fragments were purified with QiaQuick PCR extraction kit (QIAGEN, CA, USA), undergone end-repairing, dA-tailing and ligated to Illumina adapters. The ligation products were size fractioned by agarose gel electrophoresis and fragments were excised for PCR amplification to construct libraries. The libraries were sequenced using Illumina HiSeq™ 2000 (Illumina, CA, USA).

### 4.3. De Novo Assembly

The raw reads were cleaned by removing adapter sequences, low-quality sequences (reads with ambiguous bases ‘N’) and reads with more than 10% Q < 20 bases. De novo assembly of the clean reads was performed using Trinity [44] program to build the reference-free full-length transcription. In the present study, K-mer value 25 was chosen in Trinity, with group-pairs-distance =500 and other default parameters. Next, Trinity connected the contigs between each pair of contigs using ‘N’ to represent unknown bases, thus generating scaffolds. Paired-end reads were used again for scaffold gap filling to obtain sequences with the least Ns and those that could not be extended at either end. Such sequences were defined as unigenes. Finally, the potential transcript sequences were clustered using the TGI Clustering tool to obtain uni-transcripts [45].

### 4.4. Functional Annotation

To annotate the unigenes, we used the BLASTx program (http://www.ncbi.nlm.nih.gov/BLAST/) at NCBI with an *E*-value threshold of 1 × 10^−5^ to NCBI NR database (http://www.ncbi.nlm.nih.gov), the Swiss-Prot protein database (http://www.expasy.ch/sprot) and the KEGG database (http://www.genome.jp/kegg). The sequence direction of the unigenes was determined according to the best alignment results. When the results were conflicted among databases, the direction was determined consecutively by NR, Swiss-Prot and KEGG. When a unigenes could not be aligned, sequence direction would be confirmed using the ESTscan program. GO annotation was analyzed by Blast2GO software. Functional classification of the unigenes was performed using WEGO software. KEGG pathway annotation was carried out using Blastall software against the KEGG database.

### 4.5. Analysis of Differentially Expressed Genes (DEGs)

The gene expression level of all unigenes was estimated by calculating read density as reads per kilobase of exon model per million mapped reads (RPKM) [46]. The DEGs from two libraries were identified based on the Audic and Claverie’s method [47]. IDEG6 software [48] (http://telethon.bio.unipd.it/bioinfo/IDEG6_form/) was used to identify the DEGs. Results of all statistical tests were calibrated for multiple testing with the Benjamini-Hochberg false discovery rate (FDR) [49]. The FDR adjusted *p* value <0.01 and the absolute value of log_2_ Ratio >1 were used to confirm significant differences in gene expression.

### 4.6. GO and KEGG Enrichment Analysis of Differentially Expressed Genes (DEGs)

After functional annotation, the gene ontology (GO) functional classification of DEGs among the three developmental stages in FL and SL ovules was acquired. To identify the DEGs’ significantly enriched GO terms, the GO enrichment analysis of DEGs were performed. In each comparison, the gene numbers in every GO term, in which DEGs and unigenes in de novo transcriptome were mapped, were counted. The *p* value in each GO term was calculated using hypergeometric test and the corrected *p* value was further acquired using the Bonferroni correction. The GO term was considered as significantly enriched GO terms in DEGs if the corrected *p* value < 0.05.

The KEGG database was used to explore the biological pathways the DEGs involved in. After KEGG annotation, the KEGG pathway enrichment analysis was performed. In each comparison, the gene number in every KEGG pathway, that DEGs and unigenes in de novo transcriptome were mapped to, were counted. The *p* value for each pathway was acquired using hypergeometric test and the corrected *p* was calculated using the Bonferroni correction. If the corrected *p* value < 0.05, the pathway was defined as significantly enriched pathway for DEGs.

### 4.7. Real-Time Quantitative PCR Analysis

Total RNA was isolated using Plant RNA Assistant Kit (Kebaiao, Beijing, China). Reverse transcription into cDNA was conducted using SuperReal PreMix Plus (TIANGEN Biotech, Beijing, China). Quantitative real time PCR was performed using a MiniOpticon Two-Color Real-Time PCR Detection System (BIO-RAD, Hercules, CA, USA). The PCR conditions were 95 °C for 30 s, followed by 45 cycles of 95 °C for 3 s for denaturation and 60 °C for 30 s for annealing and extension. The experiments were repeated three times. Relative expression levels of the target genes were normalized and then calculated using comparative Ct (2^−ΔΔ*C*t^) method. Primers specific to the genes being characterized were designed by ‘Primer Premier 6′ (http://www.premierbiosoft.com/crm/jsp/com/pbi/crm/clientside/ProductList.jsp) with the product lengths ranging from 70–200 bp for RT-qPCR. The *EF1* and *Tubulin* genes were used as internal standard for the normalization of gene expression and the FL ovules in FNM1 stage were used as a reference sample whose value was set to 1. The relative gene expression was evaluated using the comparative cycle threshold method.

## 5. Conclusions

FNM is an indispensable process in ovule development, producing approximately 1000 free nuclei for cellularization. The transcriptome sequencing analysis presented in this study has expanded our knowledge of this process by identifying DEGs involved in crucial biological processes, such as cell division and hormone pathways. Importantly, the high-resolution expression patterns presented here highlight our understanding of the molecular mechanisms involved in the FNM process and ovule development in the conifers.

## Figures and Tables

**Table 1:** Throughput and quality of RNA-seq of the differentially expressed gene libraries.

Libraries	Total Reads	Total Nucleotides (nt)	Q20 Percentage	N Percentage	GC Percentage
FNM1-FL	12,463,236	2,517,395,209	96.70%	0.07%	45.30%
FNM1-SL	10,003,825	2,020,636,530	97.96%	0.01%	47.15%
FNM2-FL	11,556,887	2,334,321,363	96.74%	0.07%	45.31%
FNM2-SL	10,030,612	2,026,041,249	95.57%	0.01%	45.85%
FNM3-FL	15,181,332	3,066,365,571	98.54%	0.00%	45.34%
FNM3-SL	11,761,002	2,375,549,578	98.59%	0.00%	44.81%

**Table 2:** Summary of the transcriptome assembly.

Assembly Statistics	Numbers
Total number of unigenes	63,449
Mean length of unigenes (bp)	740
N50 length of unigenes (bp)	1481

**Table 3:** Summary of unigene annotation in *Pinus tabulaeformis* ovules.

Annotation Database	Number of Annotated Unigenes	Percentage of Annotated Unigenes
NR	30,766	48.5%
Swiss-Prot	22,451	35.4%
GO	25,246	39.8%
KEGG	6583	10.4%

NR, NCBI non-redundant protein database; Swiss-Prot, Swiss-Prot protein database; GO, gene ontology; KEGG, Kyoto Encyclopedia of Genes and Genomes Pathway.

**Table 4:** Differentially expressed genes (DEGs) in FL vs. SL ovules at three free nuclear mitosis (FNM) stages in FG development.

Developmental Stage	FNM1	FNM2	FNM3
Total DEGs	2203	2603	2368
Upregulated	630	900	843
Downregulated	1573	1703	1525

**Table 5:** Significantly enriched gene ontology (GO) terms in DEGs between FL and SL ovules in different stages among FNMM.

Developmental Stage	FNM1	FNM2	FNM3
BP	14	14	38
MF	3	2	15
CC	6	7	11
Total	23	23	64
Percentage	20.9%	20.9%	58.2%

BP, biological process; MF, molecular function; CC, cellular component. For a complete list of terms, see Table S3.

**Table 6:** 10 top KEGG pathways with a high representation of DEGs.

Pathways	No. of DEGs with Pathway Annotation					Pathway ID
	All Profiles (% of 1369)	Profile 0 (% of 63)	Profile 1 (% of 113)	Profile 6 (% of 98)	Profile 7 (% of 64)	
Metabolic pathways	167 (12.20%)	29 (46.03%)	42 (37.17%)	32 (32.65%)	23 (35.93%)	ko01100
Biosynthesis of secondary metabolites	96 (7.01%)	17 (26.98%)	26 (23.01%)	22 (22.45%)	8 (12.50%)	ko01110
Biosynthesis of antibiotics	33 (2.41%)	8 (12.70%)	7 (7.19%)	8 (8.16%)	1 (1.56%)	ko01130
Microbial metabolism in diverse environments	30 (2.19%)	8 (12.70%)	9 (7.96%)	1 (1.02%)	2 (3.13%)	ko01120
Phenylpropanoid biosynthesis	23 (1.68%)	3 (4.76%)	11 (9.73%)	5 (5.10%)	2 (3.13%)	ko00940
Plant hormone signal transduction	23 (1.68%)	3 (4.76%)	7 (7.19%)	4 (4.08%)	0 (0.00%)	ko04075
Carbon metabolism	22 (1.61%)	8 (12.70%)	6 (5.31%)	1 (1.02%)	1 (1.56%)	ko01200
DNA replication	20 (1.46%)	0 (0.00%)	0 (0.00%)	0 (0.00%)	16 (25.00%)	ko03030
Cell cycle-yeast	19 (1.39%)	0 (0.00%)	1 (0.88%)	0 (0.00%)	9 (14.06%)	ko04111
Biosynthesis of amino acids	18 (1.31%)	6 (9.52%)	7 (7.19%)	2 (2.04%)	0 (0.00%)	ko01230

## Supplementary Materials

Supplementary materials can be found at http://www.mdpi.com/1422-0067/19/8/2246/s1.

## Abbreviations

ABA	Abscisic acid
ABF	ABA responsive element binding factor
AFC	Average fold change
AHK	Arabidopsis histidine kinase
AHP	Arabidopsis histidine phosphotransfer protein
ARF	Auxin response factor
APC/C	Anaphase-promoting complex/cyclosome
ASA	Anthranilate synthase
AUX1	AUXIN1
BLASTX	Basic local alignment search tool X
CDC	Cell division cycle
CDK	Cyclin-dependent kinase
CKX	Cytokinin oxidases/dehydrogenases
CRE1	Cytokinin response 1
DEGs	Differentially expressed genes
EBF	EIN3-binding F-box protein
FDR	False discovery rate
FG	Female gametophyte
FL	Fertile line
FNM	Free nuclear mitosis
FNMM	Free nuclear mitosis of megagametophyte
GID	Glucose-induced degradation protein
GO	Gene ontology
ICK	CDK inhibitory protein
JAZ	Jasmonate ZIM domain-containing protein
LAX	LIKE-AUX1
MCM	Minichrome maintenance
MMC	Megaspore mother cell
MPK6	Mitogen-activated protein kinase 6
NCED	9-*cis*-Epoxycarotenoid dioxygenase
NR	Non-redundant
KEGG	Kyoto Encyclopedia of Genes and Genomes
PAT	Polar auxin transport
PP2C	Protein phosphatase 2C
PYR	Pyrabactin resistance 1 protein
PYL	PYR1-Like protein
qRT-PCR	Quantitative real-time PCR
RPKM	Reads per kb per million reads
SAUR	Small auxin-up RNA
SL	Sterile line
STEM	Short Time-series Expression Miner
