# Supplementary figures and images for: RNA-seq Analysis Reveals Gene Expression Profiling of Female Fertile and Sterile Ovules of Pinus Tabulaeformis Carr. during Free Nuclear Mitosis of the Female Gametophyte
Source: Int J Mol Sci. 2018 Aug 1;19(8):2246. doi: 10.3390/ijms19082246 (PMC6122031; doi:10.3390/ijms19082246)

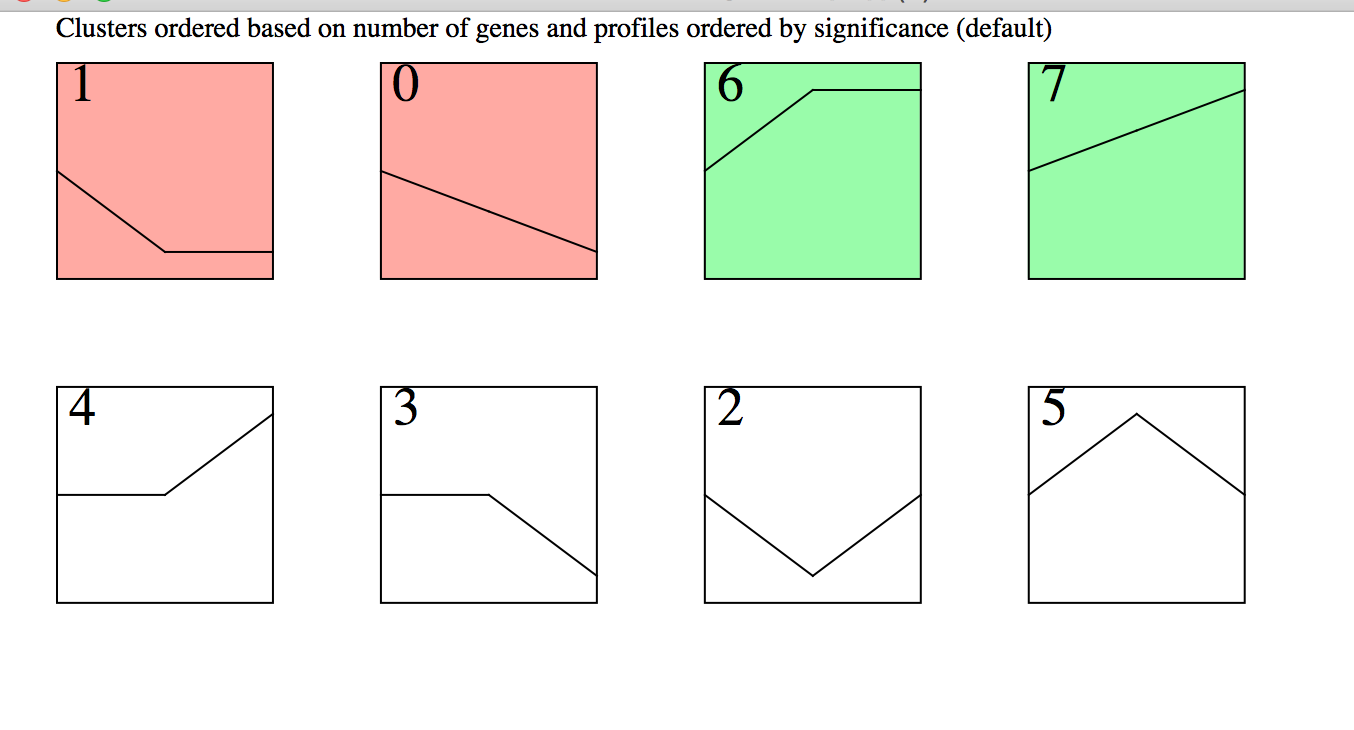

Supplement: Supplementary file 1 [file ijms-19-02246-s001.zip › Figure S5. 8 expression pattern of DEGs in FL ovules during FNMM.png]
